# Determination of Factors Associated with Natural Soil Suppressivity to Potato Common Scab

**DOI:** 10.1371/journal.pone.0116291

**Published:** 2015-01-22

**Authors:** Marketa Sagova-Mareckova, Ondrej Daniel, Marek Omelka, Vaclav Kristufek, Jiri Divis, Jan Kopecky

**Affiliations:** 1 Crop Research Institute, Dept. Epidemiology and Ecology of Microorganisms, Prague, Czech Republic; 2 Faculty of Mathematics and Physics, Dept. Probability and Mathematical Statistics, Charles University, Prague, Czech Republic; 3 Biology Centre of the Academy of Sciences of the Czech Republic, v. v. i., Institute of Soil Biology, Ceske Budejovice, Czech Republic; 4 Faculty of Agriculture, Dept. Plant Production, University of South Bohemia, Ceske Budejovice, Czech Republic; Graz University of Technology (TU Graz), AUSTRIA

## Abstract

Common scab of potatoes is a disease, which is difficult to manage due to complex interactions of the pathogenic bacteria (*Streptomyces* spp.) with soil, microbial community and potato plants. In Bohemian-Moravian Highlands in the Czech Republic two sites (Vyklantice and Zdirec) were selected for a study of common scab disease suppressivity. At both sites, a field with low disease severity occurs next to one with high severity and the situation was regularly observed over four decades although all four fields undergo a crop rotation. In the four fields, quantities of bacteria, actinobacteria and the gene *txtB* from the biosynthetic gene cluster of thaxtomin, the main pathogenicity factor of common scab, were analyzed by real-time PCR. Microbial community structure was compared by terminal fragment length polymorphism analysis. Soil and potato periderm were characterized by contents of carbon, nitrogen, phosporus, sulphur, calcium, magnesium, and iron. Quality of organic matter was assessed by high performance liquid chromatography of soil extracts. The study demonstrated that the suppressive character of the fields is locally specific. At Zdirec, the suppressivity was associated with low *txtB* gene copies in bulk soil, while at Vyklantice site it was associated with low *txtB* gene copies in the tuberosphere. The differences were discussed with respect to the effect of abiotic conditions at Zdirec and interaction between potato plant and soil microbial community at Vyklantice. Soil pH, Ca soil content or cation concentrations, although different were not in the range to predict the disease severity. Low severity of common scab was associated with low content of soil C, N, C/N, Ca and Fe suggesting that oligotrophic conditions may be favorable to common scab suppression.

## Introduction

The common scab of potatoes is an important soil-born disease with worldwide occurrence. It has been rated among the top five diseases of potatoes by seed producers in the USA [1]. The disease affects tuber quality due to superficial and pitted lesions that form around the site of infection [2]. The infection is caused by actinobacteria from the genus *Streptomyces* that possess a large (325–660 kb) pathogenicity island (PAI) in their genomes. The most important pathogenicity determinant is a phytotoxin thaxtomin synthetized from two amino-acid precursors via a condensation step catalyzed by nonribosomal peptide synthetase coded by *txtAB* genes. These genes are used for determination and quantification of pathogens responsible for the disease [3, 4, 5].

The distribution, severity and incidence of common scab have been widely studied in relationship to physico-chemical and microbial soil characteristics but the disease is still difficult to control. Traditional control strategies of common scab such as irrigation and reduced soil pH are not sufficient and often fail because the involved relationships are very complex. Several studies indicated that control measures may be soil specific [5], though it is unclear whether this is due to physical soil properties, or soil microbial flora [1].

Naturally occurring disease-suppressive soils provide a valuable resource for implementation of a sustainable alternative to current control strategies [6]. Yet more information about those soils functioning needs to be collected because different, often locally specific, mechanisms lead to soil suppressiveness. Those include situations: (i) the pathogen does not establish or persist at the site, (ii) the pathogen establishes but causes little or no damage, or (iii) the pathogen establishes and causes disease for a while but thereafter the disease ceases, although the pathogen may persist in the soil [7].

Fields suppressive to common potato scab were identified at various locations, for example in central Washington, Grand Rapids and Becker, MN, East Lansing, MI [8]. In all of those sites the suppressivity was attributed to biological interactions between antagonistic microflora and pathogens mediated via antibiotic production or enzymatic activity [9]. That type of suppressivity can establish after many years of potato (or other crop) monoculture because new protective biotic interactions are stimulated over time by continuous co-occurrence of pathogens and competing microorganisms. However, the mechanisms of suppression vary and particularly in soils undergoing regular crop rotation the suppressive character may be related to geological origin and soil physico-chemical characteristics [9].

The Bohemian-Moravian Highlands represent an important potato growing area in central Europe, where particularly seed potatoes, representing production of about 70 thousand of metric tons are produced and distributed in Europe and near East. The certification for seed potatoes requires less than 5% of surface irregularities. Soil conditions in the area do not belong to those favorable to common scab because they are usually characterized by pH between 5–6 and relatively high spring and summer precipitation with annual average about 800 mm. However, common potato scab is widely spread producing significant damage to potatoes and consequently causing economic losses.

Long history of potato growing in the area enabled to identify fields with low incidence of common potato scab. In this study, two sites Vyklantice and Zdirec where a field with low potato scab severity lies next to a field with high disease severity were selected. Those two sites undergo a regular four crop rotation, while the differences in severity of the disease between the respective fields have been constant over four decades.

The goal of the study was to determine and prove the suppressive or conducive character of the two nearby fields at each site. The approach was based on a field experiment, in which three potato cultivars susceptible to common scab were grown at the two sites (four fields) in a Latin square design with four replicates. The three cultivars were selected to account for the variability of relationships between potatoes and pathogens. The soil suppressivity or conducivity of the studied fields were determined by relationships between common scab severity, quantity of thaxtomine biosynthetic gene *txtB*, soil and periderm chemical characteristics, quality of organic matter and microbial community. Differences between those relationships determined in fields with low and high severity of common scab were used to predict mechanisms involved in disease suppressivity and to identify soil descriptors, either abiotic or biotic, associated with soil health and suitability for potato growing.

## Methods

### Ethics Statement

The field study did not involve vertebrates, endangered or protected species; it was not carried out in a protected area. The experiments were carried out on private arable land with the permission of the owners, the companies Vysocina Vyklantice a.s. (fields V_L_, V_H_) and Vesa Ceska Bela a.s. (fields Z_L_, Z_H_). No other permissions were required.

### Sites

Vyklantice and Zdirec are sites where two fields (V_L_, V_H_, and Z_L_, Z_H_, resp.) about 100m distant differ in common scab severity (marked by L and H for low and high scab severity at two sites Vyklantice, Zdirec (V, Z) by observation over 30 years. The two sites differ in environmental factors, while the two fields within each site are similar in geological context, soil type, climate and management (Table 1). All studied fields were regularly planted under four-field crop rotation system including rape, clover, potatoes, and grains (wheat, oats) in the past two decades.

**Table 1:** Site characteristics.

	**Vyklantice**		**Zdirec**	
	**V_L_**	**V_H_**	**Z_L_**	**Z_H_**
coordinates	49°33'48"N, 15°03'31"E	49°33'43"N, 15°03'18"E	49°37'37"N, 15°37'56"E	49°37'48"N, 15°37'58"E
altitude [MAMSL]	600	590	510	510
clay [%]	3	0	12	6
silt [%]	16	30	56	56
sand [%]	72	62	29	35
gravel [%]	9	8	3	3
CEC [cmol/kg]	13.3	16	15.4	20.1

### Field experiment

The field experiment was conducted in 2010. Potatoes were planted on May 27 and the samples of bulk soil, tuberosphere soil and potatoes were collected after 80 days. Three cultivars susceptible to common scab Agria, David and Valfi were used. Potatoes were all certified seed tubers (common scab below 5% surface). Four replicates of each cultivar at each field were used and arranged in a Latin square design (3 cultivars × 4 plots plus 4 bulk soils at each field and site, 16 samples per field × 4 fields). Each plot was planted with 3 rows of 12 potato plants (36 plants) separated by 50 cm of bare soil. The effect of the site and field (selectively also the effect of a cultivar) on common scab severity was assessed. At all fields, soil chemical characteristics were determined by total soil C, N, S, P, Ca, Mg, Fe content, potato chemical characteristics were determined by N, P, Ca, Mg, Fe periderm content. Microbial characteristics were described by actinobacterial diversity, quantity of bacteria, actinobacteria and thaxtomin biosynthetic gene *txtB* in both soil and potato periderm.

### Sampling

One potato plant growing in the center of each plot was sampled. Tuberosphere soil samples were collected no further than 3 mm from a potato tuber. A tuber was located by careful uncovering the top soil surrounding the plant, slightly pressed to the remaining soil and taken out. The socket remaining in soil after potato extraction was carefully scratched by a sharp spoon to collect a thin layer of soil. Soil was also collected from the tuber itself if any soil remained attached on the tuber in a thin layer. Bulk soil was collected at a distance of cca 30 cm from the closest plant within each plot using a small sterile spade. To demonstrate the effect of potato plants on microbial community, results were calculated either for all soil samples (identified “soil”) or separately for bulk or tuberosphere soil. Potatoes from the central plant were collected and washed in distilled water. All potatoes were carefully pealed by a potato peeler taking approximately 1 mm thick periderm samples, the peels were homogenized and mixed and subsamples were taken for further analyses. Common scab severity was evaluated on 20 potatoes per plot using a 9 degree scale [10]. Potatoes used for evaluation were those of the collected plant and several more plants from each plot to achieve at least 20 measurements per plot.

### Soil and potato periderm analyses

To determine total soil C, N, and S content 2-gram aliquots of homogenized soil samples from both bulk and tuberosphere were dried, milled, and analyzed using Vario MAX CNS analyzer (Elementar Analysensysteme, Hanau, Germany). To determine all other soil elements soil subsamples were leached with boiling nitro-hydrochloric acid (aqua regia) and assessed by optical emission spectroscopy with inductively coupled plasma (ICP-OES) in Aquatest company (Prague, Czech Republic). The analyses of potato periderm were performed by the service laboratory of the Institute of Botany (Trebon, Czech Republic). For total nitrogen analysis, 1–3 mg dried periderm was mineralized by modified Kjeldahl method in H_2_SO_4_ with catalyzer at 360°C. For total phosphorus analysis, 20 mg dried periderm was sequentially decomposed by HNO_3_ and HClO_4_ [11]. In the mineralized samples, both N and P were determined by flow injection analysis with spectrophotometric detection using FIA Lachat QC 8500 analyzer (Lachat Instruments, Hach Company, Loveland, CO). Cation contents in periderm were determined by atomic absorption spectrometry using AAS spectrometer ContrAA 700 (Analytik Jena, Jena, Germany) after mineralization with nitro-hydrochloric acid.

### DNA extraction

Soil samples from tuberosphere and bulk soil were homogenized and subsamples of 0.5 g were used for DNA extraction by method SK described by Sagova-Mareckova et al. [12]. Briefly, the method is based on bead-beating and phenol/chloroform extraction followed by purification with CaCl_2_ and a GeneClean Turbo kit (MP Biomedicals, Santa Ana, CA). For DNA extraction from potato periderm, 3 g of periderm samples were fine cut in sterile Petri dish, homogenized, and 0.3 g subsample was processed in the same way to obtain total periderm DNA.

### HPLC analysis of extractable low-molecular-weight soil metabolites

Soil samples for HPLC analyses were collected from the tuberosphere of Agria cultivar and bulk soil from the same plots (8 samples per field, 32 in total). Approximately 50 ml of soil were extracted with approximately 50 ml of methanol-water-acetic acid (80:19:1, vol/vol/vol) at room temperature for 1 to 2 days. The extracts were filtered and vacuum concentrated to remove organic solvent. The resulting aqueous suspension was chromatographed over Amberlite XAD 1180 (5g of aqueous suspension; diameter of glass column, 2 cm). Fifty ml of water (MilliQ quality) was used as effluent for the first (hydrophilic) fraction; 50 ml of absolute ethanol was used for the second (lipophilic) fraction. The ethanolic fraction was evaporated and diluted in methanol at a concentration of 5 mg ml^-1^. The analysis was performed using Acquity UPLC system with the 2996 PDA detector (Waters, Milford, MA). The column was a Phenomenex Luna 5 µm C18(2) 100 Å, 100 × 4.6 mm, equipped with SecurityGuard cartridge C18, 4 × 3.0 mm ID (Phenomenex, Torrance, CA). The separation was run in air-conditioned room at 20°C, and the flow rate was 1.0 ml min^-1^. Solvent A was ultrapure water; solvent B was acetonitrile. The gradient started with 10% of solvent B for 1 min and then increased linearly to 100% of B within 8 min. The final concentration was held for a further 3.5 min. Twenty µl of each sample were injected. The spectra were recorded from 194 to 800 nm in 1 nm intervals and data points recorded once per second were exported from MassLynx software (Waters) in text format for further processing.

### Terminal restriction fragment length polymorphism analysis (T-RFLP)

Soil and periderm DNA samples were analyzed by amplification of genes for 16S rRNA using universal forward primer 16Seu27f (5´-AGAGTTTGATCMTGGCKCAG-3´) [13] 5-end labelled with 6-FAM (6-carboxyfluorescein) and *Actinobacteria*-specific reverse primer 16Sact1114r (5´-GAGTTGACCCCGGCRGT-3´) [14]. Reaction mixture contained in a total volume of 50 µl: 1× polymerase buffer, 1.5 mM MgCl_2_, 400 nM of each primer, 0.2 mM of each dNTP, 0.6 mg/ml BSA, 1 U Taq polymerase (TopBio, Prague, Czech Republic). PCR amplification was performed in C1000 Thermal Cycler (Bio-Rad, Hercules, CA, USA) and the PCR program consisted of an initial denaturation at 94°C for 2 min, followed by 35 cycles of 94ºC for 45 s, annealing at 57ºC for 45 s, extension at 72ºC for 1 min 30 s, and a final extension step at 72ºC for 5 min. Labelled PCR products were purified using QIAquick PCR purification kit (Qiagen, Hilden, Germany). Labelled PCR amplicons of the 16S rRNA gene region were cleaved by *Alu*I restriction endonuclease for 4 h at 37ºC with subsequent additions of the enzyme in two equal aliquots at the beginning and in the middle. After inactivation of the restriction enzymes and purification with Sigma Spin Post-Reaction Clean-Up Columns (Sigma-Aldrich, St. Louis, MO), the samples were subjected to fragment analysis on a 96-capillary sequencer (Applied Biosystems, Foster City, CA) in Genomac company (Prague, Czech Republic). T-RFLP analyses were performed in one run for all samples. Crude profiles were filtered to the largest T-RF peaks representing 95% of the total of peak heights (areas) to eliminate the noise intermittently exceeding the cut-off limit.

### Quantitative PCR

Quantifications were performed with primers eub338f (5’-ACTCCTACGGGAGGCAGCAG-3) [15] and eub518r (5’-ATTACCGCGGCTGCTGG-3’) [16] amplifying a 197 bp fragment of the 16S rRNA gene from *Bacteria,* act235f (5’-CGCGGCCTATCAGCTTGTTG-3’) [17] and eu518r yielding 280 bp product for *Actinobacteria*, and StrepF (5’-GCAGGACGCTCACCAGGTAGT-3’) and StrepR (5’-ACTTCGACACCGTTGTCCTCAA-3’) yielding 72 bp amplicon of the thaxtomin biosynthetic gene *txtB* [18], respectively. The analyses were done on a StepOne Plus Real-Time PCR System (Applied Biosystems, Foster City, CA) using 96-well plates with GoTaq qPCR Master Mix (Promega, Madison, WI) containing SYBR Green as a double-stranded DNA binding dye. The reaction mixture contained in a total volume of 15 µl: 1× GoTaq qPCR Master Mix, 0.2 µM primers, and 0.2–2 ng diluted DNA sample. For all of the mentioned targets the PCR cycling protocol consisted of initial denaturation at 95°C for 10 min, followed by 45 cycles of 95°C for 15s, 60°C for 30s and 72°C for 30s. Melting curves were recorded to ensure qPCR specificity. Baseline and threshold calculations were performed with the StepOne v. 2.2.2 software. The inhibition was tested by serial DNA dilution from each site. All qPCR measurements were done in duplicate.

The qPCR standards were prepared by cloning the fragments of the target genes in pGEM-T Easy vector system (Promega). After PCR verification and isolation of the cloned constructs by Pure Yield Plasmid Miniprep System (Promega), a linear standard was prepared by cleaving with *Sal*I enzyme (New England Biolabs, UK) in a 200 µl reaction mixture containing 1× reaction buffer, 2 µg circular plasmid, and 20 U restriction endonuclease for 2h in 37°C. The linearized plasmid DNA was purified by phenol-chloroform extraction. The aliquots of linearized and purified standard diluted to 20 ng/µl were stored in-70°C.

### Data analysis

The differences between suppressive and conducive fields were tested by ANOVA and Welch’s two sample *t*-test (allowing differences between variability of variables), which aims at detection of differences in mean values. All variables were log-transformed to make their distribution more similar to a normal distribution. The HPLC absorption signals recorded at the wavelength 210 nm were baseline corrected and normalized by division through the median of the absorption values. For the T-RFLP and HPLC profiles, the Manhattan metric (sum of absolute differences) was used to calculate the distance matrices. The distance matrices were plotted by Sammon’s Multidimensional Scaling [19]. Two tests based on distance matrices was used. While PERMANOVA [20] tests that the within group distances are on average shorter than the between group distances (aiming at detection of different mean profiles), dispersion test (denoted later as ‘disp’) introduced in Gijbels and Omelka [21] tests that the average within group distances differ among the groups (aiming at detecting of different dispersions of the samples). The correlation coefficients at different fields were compared through the permutation test introduced in Omelka and Pauly [22]. All statistical calculations were done in the R-computing environment [23].

## Results

### Common scab

At both sites, Vyklantice (V) and Zdirec (Z), the two fields (V_L_, V_H_, and Z_L_, Z_H_) differed significantly in common scab severity (V_L_ x V_H_ p < 0.001, Z_L_ x Z_H_ p < 0.001) (Table 2). The fields V_H_ and Z_H_ had higher common scab severity than the respective fields V_L_ and Z_L_ at the same site (Table 2).

**Table 2:** Biological characteristics.

		**VL**		**VH**		**ZL**		**ZH**		**p-values**		
	**N**	**mean**	**S.E.**	**mean**	**S.E.**	**mean**	**S.E.**	**mean**	**S.E.**	**overall ANOVA**	**VL vs. VH**	**ZL. vs. ZH**
Soil												
bacteria	16	10.30	0.06	10.10	0.04	10.10	0.06	10.20	0.04	0.073	0.059	0.058
actinobacteria	16	9.57	0.05	9.53	0.03	9.57	0.03	**9.75**	0.04	0.001	0.534	0.001
*txtB*.total (bulk and tuberosphere)	16	4.38	0.07	4.49	0.12	4.93	0.19	**5.57**	0.14	< 0.001	0.436	0.013
*txtB*.bulk	4	**4.53**	0.04	3.97	0.07	4.00	0.22	**4.93**	0.06	< 0.001	0.002	0.02
*txtB*.tuberosphere	12	4.33	0.09	**4.67**	0.13	5.80	0.14	**5.27**	0.15	< 0.001	0.044	0.015
Periderm												
actinobacteria	12	7.82	0.12	**8.74**	0.17	9.40	0.13	9.27	0.11	< 0.001	< 0.001	0.45
*txtB*.periderm	12	6.10	0.05	**7.18**	0.16	7.31	0.15	7.51	0.12	< 0.001	< 0.001	0.312
Common scab severity	12	2.34	0.14	**4.06**	0.14	4.23	0.15	**6.65**	0.15	< 0.001	< 0.001	< 0.001

Quantity of bacteria, actinobacteria (16S rRNA gene) and *txtB* gene in soil and periderm. Means and respective standard errors (gene copies per gram soil, log10 transformed). Significantly higher values are in bold.

### Soil and periderm biological characteristics

In bulk soil, the field V_L_ had significantly higher *txtB* gene copies than V_H_ (p = 0.002), while Z_L_ had significantly lower *txtB* gene copies than Z_H_ (p = 0.020) (Table 2). In tuberosphere soil, the field V_L_ had significantly lower *txtB* gene copies than the field V_H_(p = 0.003), while Z_L_ had significantly higher *txtB* gene copies than Z_H_ (p = 0.015). In periderm, the field V_L_ had significantly lower *txtB* gene copies than the field V_H_ (p < 0.001), while Z_L_ did not differ from Z_H_. Also, the field V_H_ had significantly higher actinobacteria in soil than the field V_L_, while the field Z_H_ had significantly higher actinobacteria in periderm than the field Z_L_ (Table 2).

### Soil and periderm chemical characteristics

The field V_H_ had significantly higher pH and contents of soil (bulk and tuberosphere) C, N, P, Ca, Fe and periderm P, Ca, Fe, while it had significantly lower content of soil Mg than the field V_L_. The field Z_H_ had significantly higher pH and contents of soil (bulk and tuberosphere) C, N, S, Mg, Ca, Fe and periderm Ca, while it had significantly lower soil P than the field Z_L_ (Table 3).

**Table 3:** Chemical characteristics in soil and periderm.

		**VL**		**VH**		**ZL**		**ZH**		**p-values**		
	**N**	**mean**	**S.E.**	**mean**	**S.E.**	**mean**	**S.E.**	**mean**	**S.E.**	**overall ANOVA**	**VL vs. VH**	**ZL. vs. ZH**
Soil												
pH	16	5.61	0.03	**6.43**	0.03	6.87	0.03	**7.01**	0.03	< 0.001	< 0.001	< 0.001
C (%)	16	1.28	0.03	**2.04**	0.03	1.67	0.04	**2.33**	0.05	< 0.001	< 0.001	< 0.001
N (%)	16	0.146	0.003	**0.214**	0.003	0.178	0.003	**0.221**	0.003	< 0.001	< 0.001	< 0.001
S (mg.kg^-1^)	16	744.0	23.1	702.0	16.8	244.0	6.5	**305.0**	3.3	< 0.001	0.151	< 0.001
P (mg.kg^-1^)	16	921.0	13.8	**1180.0**	12.8	**858.0**	20.3	722.0	7.4	< 0.001	< 0.001	< 0.001
Mg (g.kg^-1^)	16	**12.40**	0.22	11.80	0.18	3.78	0.18	**4.56**	0.06	< 0.001	0.043	0.001
Ca (g.kg^-1^)	16	1.89	0.10	**3.20**	0.13	2.34	0.11	**4.15**	0.10	< 0.001	< 0.001	< 0.001
Fe (g.kg^-1^)	16	43.00	0.63	**46.80**	0.87	22.50	0.89	**27.10**	0.38	< 0.001	0.001	< 0.001
Periderm												
N (g.kg^-1^)	12	23.40	1.01	25.80	0.73	23.30	0.83	23.50	0.80	0.105	0.068	0.835
P (g.kg^-1^)	12	1.95	0.10	2.19	0.12	2.71	0.10	3.18	0.20	< 0.001	0.136	0.054
Ca (mg.kg^-1^)	12	0.57	0.06	**1.04**	0.10	0.68	0.07	**1.00**	0.09	< 0.001	0.001	0.008
Mg (mg.kg^-1^)	12	1.09	0.03	1.04	0.02	1.12	0.03	1.07	0.04	0.264	0.146	0.433
Fe (mg.kg^-1^)	12	0.37	0.03	**0.51**	0.05	0.70	0.09	0.71	0.15	0.007	0.035	0.966

Means and respective standard errors. Significantly higher values are in bold.

### Actinobacterial communities

**Figure 1:**
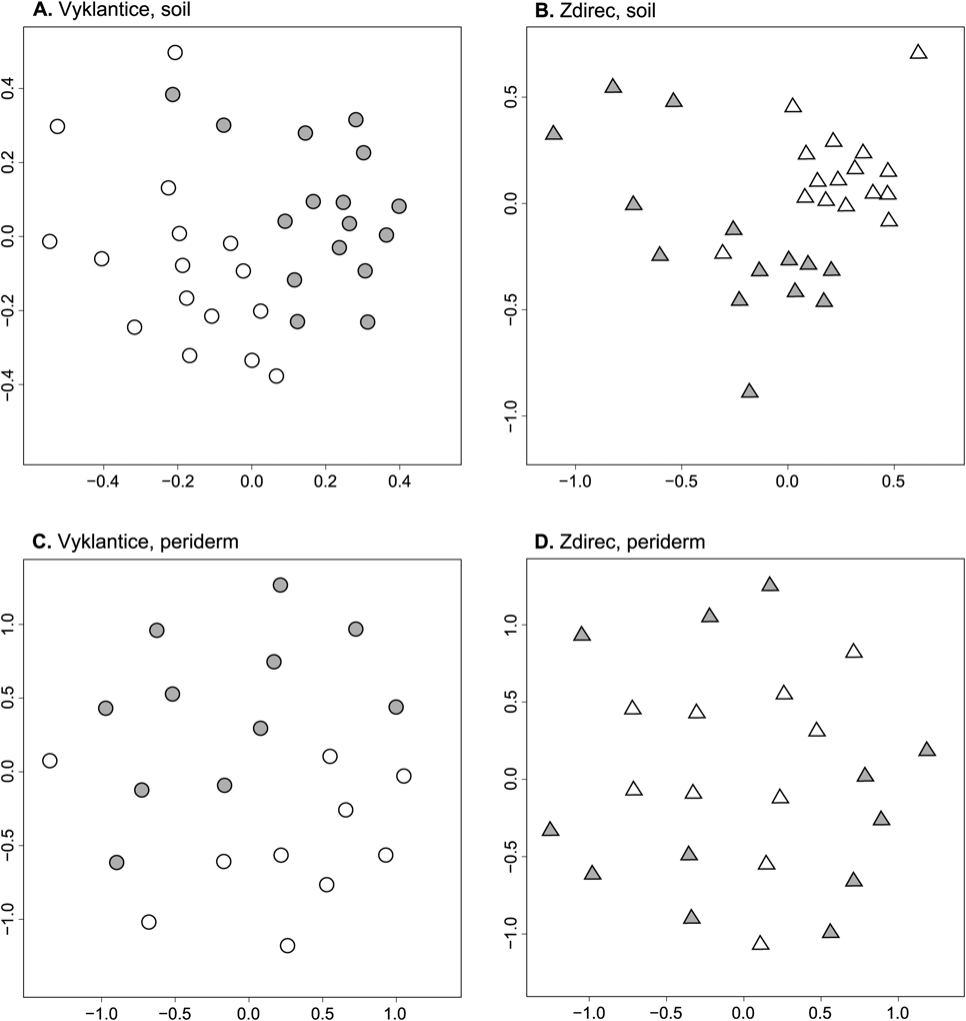
T-RFLP profiles of the communities of actinobacteria sampled at Vyklantice and Zdirec experimental sites from tuberosphere soil (A,B) and potato periderm (C,D). Sammon’s multidimensional scaling was based on Manhattan distance matrices calculated for the baseline filtered and normalized T-RFLP profiles. The symbols represent the profiles of actinobacteria at the fields with low (open symbols) and high (closed symbols) common scab severity.

T-RFLP profiles of actinobacteria in soil samples differed significantly between sites Vyklantice and Zdirec (p-permanova < 0.001, p-disp = 0.003, not presented in a figure) and between the fields within each site (Fig. 1; p-permanova < 0.001 both sites, p-disp = 0.042 Zdirec). T-RFLP profiles of actinobacteria in periderm samples did not differ between sites Vyklantice and Zdirec but differed significantly between fields (Vyklantice: p-permanova = 0.009, Zdirec: p-permanova = 0.019 and p-disp = 0.017). T-RFLP profiles of periderm samples were significantly different between cultivars at both sites (Vyklantice: p-disp = 0.005; Zdirec: p-permanova = 0.022; not presented in a figure).

### Low-molecular-weight compounds

**Figure 2:**
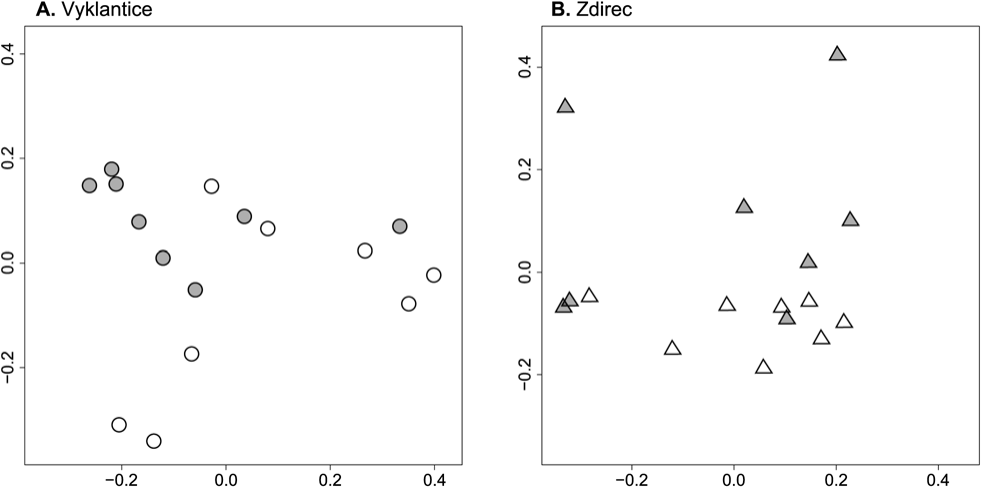
HPLC-UV profiles of extractable low-molecular-weight compounds at Vyklantice (A) and Zdirec (B) sites. Sammon’s multidimensional scaling was based on Manhattan distance matrices calculated from normalized signal at 210 nm (for details, see Materials and Methods). The symbols represent the profiles of compounds extracted from the fields with low (open symbols) and high (closed symbols) common scab severity.

The HPLC profiles of extractable low-molecular-weight compounds of soil samples were significantly different between sites (p-permanova = 0.009) and fields in Vyklantice (p-permanova = 0.033) and Zdirec (p-disp = 0.049) (Fig. 2).

## Discussion

Common scab severity differed between the two fields at each site, while the pathogen identified by the thaxtomin biosynthetic gene *txtB* was always present. Therefore, we considered the fields with low common scab severity suppressive to the disease. Both proposed suppressive fields represent the long-term/natural type of disease suppressivity functioning over long time in spite of regular crop rotation [24].

This type of suppression is likely a function of the broad physical and chemical characteristics of the soil not only of soil microbial communities [25]. In our study, the fields with higher severity of common scab had in common significantly higher content of soil C, N, C/N, Ca, Fe and higher pH. High soil pH and Ca were associated with high disease severity in other studies [5, 26, 27] but it was demonstrated that even this relationship was site specific. The observed threshold of pH for low disease soils was established by Lacey et al. [28] as a limit to common scab severity below 5.0–5.2 but the soil pH of all studied fields was above this value. Also, common scab was not observed on potatoes grown in soil with combined exchangeable Ca, Mg and K at 12 cmol/kg or less [28] but in our fields only the conducive soil of the Z_H_ field had cation concentration above this value. So, the lower soil pH or cation concentration were not likely the cause of suppressivity in our fields with low disease severity.

The two suppressive fields at both sites had lower Ca in periderm, the field in Vyklantice also periderm Fe. For accumulation of Ca in plant cells induced by thaxtomin it was established that increase in infected cells is rather an effect than a cause of infection because this toxin induced a rapid Ca^2+^ influx and cell death in *Arabidopsis thaliana* cell suspensions [29]. Fe ions represent one of redox transformations brokers [30]. Increased iron may be a result of cross talk between iron and manganese, iron and zinc, manganese and phosphate, and copper and zinc, among the many documented interactions, but may be also related to reactive oxygen species (for iron and manganese) as a result of pathogen stress [30]. Since Ca is closely related to soil pH and Fe may be rather a result of infection, just low soil C, N, and C/N remain potentially associated with suppressivity to common scab at our sites. That result was complemented by specifically high soil Mg and P contents in the suppressive fields at Vyklantice and Zdirec, respectively.

Soil organic matter (SOM) can enhance disease-suppressive activities of soil microbial communities [31–33]. In our study, soil organic matter content was high in conducive soils but the HPLC profiles of low molecular weight compounds differed between the fields with proposed suppressivity. Therefore it showed that quality rather than quantity of SOM was related to suppressivity in our fields.

Soil actinobacterial communities were significantly different between the two fields with low and high common scab severity at both sites. That is consistent with previous findings at similar disease suppressive sites, where differences between microbial community structures were assigned the primary cause of disease suppressivity [6, 8]. The T-RFLP method which was used here can demonstrate general differences between actinobacterial communities so we propose that the determined differences must be due to many taxa at various taxonomic levels. The suppressive character of soils was often associated with multiple species [6, 8, 34]. Yet, since soil microbial communities are site specific [35, 36] it may be difficult to show the taxa particularly responsible for soil suppressivity [8, 34].

Periderm actinobacterial communities differed significantly between the fields and potato cultivars at both sites but not between the two sites. That showed the importance of the effect of cultivars in spite of the differences in the soil microbial communities, which was found previously [37]. It was also proposed that the cultivar distinctive microbial community may be related to cultivars’ specific traits including resistance to pathogens [38, 39].

In our study it seemed that the character of suppressivity differed between the two sites. Firstly, the bulk soil at the suppressive field in Zdirec (Z_L_) had lower number of *txtB* gene copies than the conducive field (Z_H_), while in Vyklantice the suppressive field (V_L_) had higher number of *txtB* gene copies than the conducive field (V_H_). Secondly, the tuberosphere was the opposite because the suppressive field in Zdirec (Z_L_) had significantly higher txtB gene copies than the conducive field (Z_H_), while the suppressive field in Vyklantice had significantly lower txtB gene copies than the conducive field (V_H_). Therefore it seemed that at Zdirec the soil conditions directly suppressed the pathogen development because in the suppressive field it was low in bulk soil but increased in the tuberosphere. This may be possibly related to increased phosphorus concentration in the suppressive soil there because P content was previously often associated with low disease severity [40, 41]. In the contrary at Vyklantice the interaction between the potato plant and microbial community may be responsible for the suppression because the pathogen populations decrease in the tuberosphere. Different microbial taxa were found to participate in suppression of diseases either directly or indirectly by enhancing plant nutrition by acquiring limiting nutrients or producing beneficial enzymes (e.g. [24, 42]) and consequently supporting the plant self-protection capabilities. Also actinobacteria, namely nonpathogenic streptomycetes, were suggested to participate in common scab suppression [43].

In conclusion, we demonstrated that indeed the suppressive character of fields is locally specific. In our study it seemed that soil pH, calcium content or cation concentrations were not predicting the disease severity. Microbial communities had a site specific character corresponding to the particular field in soil and corresponding to potato cultivar in periderm. Regarding the chemical soil characteristics it seemed that oligotrophic conditions possibly complemented by specific quality of organic matter are favorable to disease suppression because C, N, Ca and Fe were less in soils with low common scab severity. The ratio between the number of *txtB* gene copies in soil and common scab severity was a good indicator of suppressivity. In the future studies, the effect of soil P and Mg on suppression of the disease in the respective situations may be applied to evaluate the use in management of the disease severity.

## Supporting Information

S1 Table Chemical and biological properties of the bulk soil, potato tuberosphere and periderm at Vyklantice site. (XLSX)

S2 Table Chemical and biological properties of the bulk soil, potato tuberosphere and periderm at Zdirec site. (XLSX)

S3 Table Common scab severity. (XLSX)

S4 Table T-RFLP profiles of actinobacterial communities in the bulk soil and potato tuberosphere at Vyklantice site. (XLSX)

S5 Table T-RFLP profiles of actinobacterial communities in potato periderm at Vyklantice site. (XLSX)

S6 Table T-RFLP profiles of actinobacterial communities in the bulk soil and potato tuberosphere at Zdirec site. (XLSX)

S7 Table T-RFLP profiles of actinobacterial communities in potato periderm at Zdirec site. (XLSX)

S8 Table HPLC profiles of extractable low-molecular-weight substances in the bulk soil and potato tuberosphere. (XLSX)
